# Radon-induced lung cancer deaths may be overestimated due to failure to account for confounding by exposure to diesel engine exhaust in BEIR VI miner studies

**DOI:** 10.1371/journal.pone.0184298

**Published:** 2017-09-08

**Authors:** Xiaodong Cao, Piers MacNaughton, Jose Cedeno Laurent, Joseph G. Allen

**Affiliations:** Department of Environmental Health, Harvard T.H. Chan School of Public Health, Boston, Massachusetts, United States of America; Stony Brook University, Graduate Program in Public Health, UNITED STATES

## Abstract

**Background:**

EPA reported that radon is the second leading cause of lung cancer in the United States, killing 21,100 people per year. EPA relies on the BEIR VI models, based on an evaluation of radon exposure and lung cancer risk in studies of miners. But these models did not account for co-exposure to diesel exhaust, a known human carcinogen recently classified by IARC. It is probable then that a portion of the lung cancer deaths in the miner cohorts are originally attributable to the exposure to diesel rather than radon.

**Objective:**

To re-evaluate EPA’s radon attributable lung cancer estimates accounting for diesel exposure information in the miner cohorts.

**Methods:**

We used estimates of historical diesel concentrations, combined with diesel exposure-response functions, to estimate the risks of lung cancer attributable to diesel engine exhaust (DEE) exposure in the miner studies. We re-calculated the fatal lung cancer risk attributable to radon after accounting for risk from diesel and re-estimated the number of U.S. deaths associated with radon in the U.S. using EPA’s methodology.

**Results:**

Considering the probable confounding with DEE exposure and using the same estimate of baseline mortality from 1989–91 that the EPA currently uses in their calculations, we estimate that radon-induced lung cancer deaths per year are 15,600 (95% CI: 14,300, 17,000)– 19,300 (95% CI: 18,800, 20,000) in the U.S. population, a reduction of 9%–26%. The death estimates would be 12,900–15,900 using 2014 baseline vital statistics.

**Conclusions:**

We recommend further research on re-evaluating the health effects of exposure to radon that accounts for new information on diesel exhaust carcinogenicity in BEIR VI models, up-to-date vital statistics and new epidemiological evidence from residential studies.

## Introduction

Long-term exposure to radon is considered to be the second most frequent cause of lung cancer after cigarette smoking [1]. The U.S. Environmental Protection Agency (EPA) estimates that individuals have a 23 in 1000 (10^−3^) risk of dying from lung cancer after a lifetime of exposure to indoor radon at EPA’s current action level of 4 picoCuries per Liter of air (pCi/L) [2]. For smokers, the risk is an extraordinary 62/1000, or over 6%. This is an uncharacteristically high risk for environmental exposure action levels; EPA normally regulates to keep risk limits at 1 in 100,000 (10^−5^) or 1 in 1,000,000 (10^−6^) [3]. Because of this high unit risk level, and widespread exposure to radon, EPA estimates that 21,100 people in the U.S. die of lung cancer every year [2]. As such, significant resources are dedicated to educating and controlling radon exposures; EPA’s FY2015 budget for state radon activities is $8.1 million [4].

The EPA’s risk estimates for radon are based on the models initially created by the National Research Council’s Committee on the Health Effects of Exposure to Radon, BEIR VI [5]. BEIR VI relied on 11 cohort studies of workers in mines to derive their risk estimates for radon. The committee developed risk models based on a combined statistical analysis of epidemiologic results from the 11 cohorts, including 2,700 lung cancers among 68,000 miners, from nearly 1.2 million person-years of observations. The models were projected to estimate risks from residential exposures without modification, which was supported by a later EPA-sponsored study [6].

Considering the high risk associated with radon exposure derived from the occupational cohorts, and the knowledge that radon and its progeny (short-lived decay products) are ubiquitous in built environments and can accumulate to harmful levels in homes and workplaces, observing effects in the general population should be possible. Residential studies yield the most relevant risk estimates to the population of interest. Turner et al. [7] studied the radon risks based on the American Cancer Society Cohort Prevention study from 1982 to 1988. They observed a 0.15 (95% CI: 0.01, 0.31) increase in the risk of lung cancer death per 100 Bq/m^3^ (2.7 pCi/L) increase in radon. Darby et al. [8] reported a collaborative study of 13 European studies indoors, including 7,148 cases of lung cancer and 14,208 controls. They found a significant linear dose-response relation at residential exposure. The relative risk of lung cancer increased by 0.16 (95% CI: 0.05, 0.31) per 100 Bq/m^3^ increase in radon exposure concentration, quite consistent to the results of Turner et al. [7]. The relation was linear with no threshold observed for radon concentrations below 200 Bq/m^3^ (5.4 pCi/L). A Germany pooled study [9] showed a meaningful radon risk at a level of 140 Bq/m^3^ (3.8 pCi/L), close to EPA’s current action level. Krewski et al. [10, 11] performed combined analysis of seven primary North American case-control studies with a total of 3,662 cases and 4,966 controls. They reported an odds ratio (OR) of 1.10 (95% CI: 1.00, 1.28) after exposure to radon at a concentration of 100 Bq/m^3^ within the exposure time window 5–30 years prior to the index date. It was compatible with the estimates of 1.12 (95% CI: 1.02, 1.25) per 100 Bq/m^3^ downwardly extrapolated from the miner data. Collectively, these residential studies have provided robust evidences of association between residential radon exposure and lung cancer risk.

However, some other studies showed null finding between residential radon exposures and lung cancer incidence. Two case-control studies in the U.S. failed to show a statistically significant link between residential radon exposure and the risk of lung cancer [12, 13]. Wilcox et al. [12] studied the radon risks in five counties in New Jersey involved 561 cases and 740 controls. The adjusted excess odds ratio (EOR) per 100 Bq/m^3^ was -0.13 (95% CI: -0.30, 0.44) for males, 0.29 (95% CI: -0.12, 1.70) for females and 0.05 (95% CI: -0.14, 0.56) for all subjects. But these associations were not statistically significant. A German case-control study [14] also failed to show a potent link between radon and lung cancer for non-smoking women, including 234 cases and 535 controls. However, their one-year radon measurements were only completed for 58% of the cases and 84% of the controls. In these studies, the uncertainties in radon measurement and exposure history may have resulted in underestimation of the true exposure-response relationship.

EPA and BEIR VI committee also reviewed some early population-based epidemiological studies and excluded them from their estimates of radon risk, deciding to solely rely on the cohort studies of miners. Their basis for excluding these studies is that the risk estimates obtained from those studies are inconsistent and imprecise, because of the very low exposures at residential level. Furthermore, the residential studies offer little opportunity for studying the modifying effects of factors such as smoking and time since exposure. Though newer residential studies have already provided more direct evidence of the dose-response relations of radon risks for both smokers and non-smokers, they were not yet incorporated into the EPA’s risk assessment.

The approach solely relied on miner data has important limitations, fully acknowledged by the BEIR VI committee. Only men of working age are included in the miner cohorts, yet the derived risk estimates from the working men are applied to women, children, and non-working men. In addition, the radon doses in the mines were very high, often more than 10 times the lifetime exposure typically found in homes (about 14 WLM), requiring low dose extrapolation to derive population risk estimates. Another critical limitation is the possible confounding with other exposure in mines, e.g. diesel exhaust.

Diesel engine-powered equipment have been widely used in trucking, railroad and underground mining facilities. The diesel engine exhaust (DEE) contains a mixture of gases and small soot particles consisting of elemental carbon. Respirable elemental carbon (REC) is the main component of DEE, and frequently used as a surrogate of the exposure to DEE. The generated diesel soot particles are readily respirable, and are associated with adverse health effects, including cardiovascular and respiratory disease, and lung cancer. Underground miners are exposed to DEE primarily from ore extraction, haulage, and transport vehicles. Mine Safety and Health Administration (MSHA) currently enforces diesel particulate matter standards at underground metal/nonmetal mines. Today, a miner's personal exposure to diesel particulate matter must not exceed 160 μg/m^3^ of total carbon when measured as an 8-hour time-weighted average [15]. Yet these exposure limits did not exist at the time represented by the 11 miner studies.

Similar to radon, a recent study showed that the unit risk estimate for diesel exhaust is strikingly high at 2.1 excess lung cancer deaths per 1,000 individuals for a lifetime exposure at an environmental concentration of 0.8 μg/m^3^ [16]. In 2012, the National Institute for Occupational Safety and Health (NIOSH) and the National Cancer Institute (NCI) completed a retrospective exposure assessment for over 12,000 mine workers at eight non-metal mining facilities (three potash, three trona, one limestone, and one salt), known as the Diesel Exhaust in Miners Study (DEMS) and report REC concentration. In these mines, the first year of the dieselization ranged from 1947 to 1967, depending on the facility [17]. For the time period of the miner cohorts used by BEIR VI and EPA (1950s to 1970s), historical REC concentrations were typically in the range of 0–600 μg/m^3^ [17]. At the time they developed the radon risk models, both BEIR VI committee and EPA listed DEE exposure as a suspected confounder to the estimates of the risk of radon progeny. However, they concluded that DEE appeared to be a weak carcinogen and probably not a necessary modifier [5]. Later, several epidemiological studies supported that occupational DEE exposure elevated the lung cancer risk in both underground non-metal mines [18] and trucking industries [19, 20]. In 2012, a working group of the International Agency for Research on Cancer (IARC) reviewed the carcinogenic effect of DEE and classified DEE as a known human carcinogen (Group 1) [21].

It is probable then, and even highly likely, that a portion of the lung cancer deaths in the miner cohorts are originally attributable to the exposure to DEE rather than radon, meaning that EPA’s risk estimates for radon are erroneously inflated. To test this assumption, we estimated the potential risks attributable to DEE exposure in the 11 miner cohorts using recently published DEE exposure-response functions [16] and a plausible DEE range in the mines based on historical data. We then applied EPA’s methodology to re-calculate the estimated annual lung cancer deaths attributable to radon after accounting for potential diesel exposure in the mines.

## Materials and methods

### Lung cancer risks from radon exposure in the 11 miner cohorts

The excess relative risks (ERR) of lung cancer reportedly follow a linear relation to the exposure of radon, which has been justified by the 11 miner cohort studies [22] and 13 European case-control studies [8]. Table 1 shows the key information on the epidemiological studies of the 11 miner cohorts relied on by BEIR VI. Exposures are measured in units of working level months (WLM). The risk estimates for each of the 11 miner cohorts (*i)* relied on by BEIR VI could be calculated by a simple linear model with a constant ERR per exposure [5]:

$$ERR_{radon,i}=\beta_{radon,i}w_{i}$$ (1)

Where *β*_*radon*, *i*_ is the estimated cohort-specific exposure-response coefficient (ERR/WLM); and *w*_*i*_ is the cohort-specific cumulative radon-progeny exposures. As shown in Table 1, the magnitude of *β*_*radon*, *i*_ varied considerably across the cohorts, ranging from 0.16 to 5.06 per 100 WLM.

**Table 1:** Summary information of the radon exposures in the 11 miner cohort studies.

Cohort	Metal type	Mean first year exposed	Mean duration (y)	Mean radon exposure (WLM)^a^^,^ ^c^	Mean concentration (WL)^a^^,^ ^c^	*β*_*radon*, *i*_ (%)
China	Tin	1955.6	12.9	286.0	1.7	0.16
Czechoslovakia	Uranium	1951.0	6.7	196.8	2.8	0.34
Colorado^b^	Uranium	1953.0	3.9	578.6	11.7	0.42
Ontario	Uranium	1963.8	3.0	31.0	0.9	0.89
Newfoundland	Fluorspar	1954.1	4.8	388.4	4.9	0.76
Sweden	Iron	1934.1	18.2	80.60	0.4	0.95
New Mexico	Uranium	1965.6	5.6	110.9	1.6	1.72
Beaverlodge (Canada)	Uranium	1962.6	1.7	21.2	1.3	2.21
Port Radium (Canada)	Uranium	1952.3	1.2	243.0	14.9	0.19
Radium Hill (Australia)	Uranium	1956.0	1.1	7.6	0.7	5.06
France	Uranium	1956.8	7.2	59.4	0.8	0.36
Total		1954.0	5.7	164.4	2.9	

^a^Weighted by person-years; includes 5-year lag interval.

^b^Exposures limited to < 3,200 WLM.

^c^One working level (WL) is defined as any combination of short-lived radon progeny per liter of air that releases 1.3×10^5^ million electron volts of alpha energy in decay. The ventilation was often poor in old uranium mines, and the radon progeny was approximately equilibrium with the radon itself. Under these conditions, each WL of radon progeny would correspond to 100 pCi/L of radon in air. Exposure to 1 working level (WL) for 170 h is defined as 1 working level months (WLM).

Based on a combined analysis of the 11 miner cohorts, BEIR VI committee further developed two extended risk models: the exposure-age-duration (EAD) model and the exposure-age-concentration (EAC) model. A 5-year latency period for the cumulative exposure was incorporated. Mathematically, the ERR calculated by the two models can be represented as:

$$ERR_{radon}=\beta_{radon,EAD\left(C\right)}\left(w_{5-14}+\theta_{15-24}w_{15-24}+\theta_{25+}w_{25+}\right)\emptyset_{age}\gamma_{z}$$ (2)

Where *β*_*radon*, *EAD(C)*_ is the exposure-response coefficient for the EAD or EAC model (ERR/WLM); the risk factors *w*_*5–14*_, *w*_*15–24*_ and *w*_*25+*_ define the cumulative radon-progeny exposures incurred in 5–14, 15–24 and more than 25 years prior to the attained age, respectively; *θ*_*15–24*_ and *θ*_*25+*_ represent the relative contributions to risk from exposures 15–24 y and 25+ y before the attained age; the risk factors *φ*_*age*_ and *γ*_*z*_ denote the modifying effects of the attained age, and either the exposure duration (in the EAD model) or exposure rate (in the EAC model). Specifically, the exposure-response relation (ERR/WLM) decreases with time since exposure, attained age, and exposure rate, but increases with exposure duration, known as the “inverse dose rate” effect. In their regressions, these risk factors were constrained to be the same in all cohorts. However, the risk coefficient was allowed to vary considerably among the cohorts. The linear regression method was used to estimate the *β*_*radon*, *EAD(C)*_. BEIR VI reported the fitted *β*_*radon*, *EAD*_ as 0.0055 and *β*_*radon*, *EAC*_ as 0.0768, based on the data from all the cohorts. More details of these models can be found in the BEIR VI report [5].

### Lung cancer risks from DEE exposure

Silverman et al. [18] studied the exposure-response relation between DEE and lung cancer quantitatively based on the historical data in the previously-mentioned eight non-mental mining facilities. They found statistically-significant positive relationship between the odds ratios of lung cancer risks and the cumulative exposures to REC, after the adjustment for smoking and other potential confounders. Their results showed a rapid increase in risks with increasing exposures at low-to-moderate levels followed by a plateauing of risks among high exposed subjects. Recently, Vermeulen et al. [16] further developed a log-linear relative risk model between DEE and lung cancer based on the data derived from three key occupational cohorts [18–20]:

$$lnRR_{DEE}=\beta_{DEE}\left(DEE\;dose\right)+\beta_{0}$$ (3)

Where DEE dose is an abbreviation for the cumulative exposure to REC in μg/m^3^-years; *β*_*DEE*_ is the combined risk coefficient for the DEE exposure across studies; *β*_*0*_ is the random intercept, which can be ignored in the risk calculation. The fitted *β*_*DEE*_ is 0.00098 (95% CI: 0.00055, 0.00141) with a significant p-value of 0.002.

### Estimating DEE concentrations in the 11 miner cohorts

To our knowledge, historical REC data are not available for the 11 miner studies to calculate the RR_DEE_. To address this, we set an exposure range based on the available REC data in other underground mining facilities that operated during the same time period, accounting for the rapidly changing use of diesel-generating equipment in mines at that time (see Table 1). The historical REC concentration in the eight non-metal mining facilities has been systematically studied by Vermeulen et al. [17] and Steward et al. [23] from 1950–2000. Their estimations were preferred by NIOSH/NCI for epidemiological analyses of the relationship between diesel exposure and lung cancer risk in these mines. According to their estimation, the REC concentration was essentially zero in the 1950s because diesel generating equipment was not widely used, and started to increase slowly in 1950s. The REC concentration was in the range of 20–60 μg/m^3^ in 1960 and 110–350 μg/m^3^ in 1970, after excluding the maximum and zero values. That indicated a rapid growth in using diesel engine-powered equipment during 1960s.

As shown in Table 1, eight out of the eleven miner cohorts worked in uranium-mining facilities with an average first year exposed of 1954. Diesel engine-powered equipment were commonly used in the uranium and hard-rock mining operations [24]. It is certainly know that diesel engine-powered equipment have been used in the New Mexico, Ontario and Colorado mines, and not in the Sweden and Beaverlodge mines [24, 25]. Most of the exposure period in the Sweden mine was before 1950, when diesel engine-powered equipment have not been used in mining operations yet. Therefore, we assumed that no DEE exposure existed in the Sweden and Beaverlodge mine in our analysis.

We set the historical REC level in the other 9 mines based on the concentration range provided by Vermeulen et al. [17]; the REC concentration range in each year can be obtained by linear interpolation. Finally, the cohort-specific RR_DEE_ were calculated with the corresponding DEE dose range during the same time period of the radon exposure in each cohort separately, as shown in Table 2.

**Table 2:** Modified *β*_*radon*, *i*_ in the 11 miner cohorts.

Cohort	DEE dose (μg/m^3^-years)		RR_DEE_ (95% CI)		Modified *β*_*radon*, *i*_ (%) (95% CI)^a^^,^ ^b^			
	Lower limit	Upper limit	Lower limit	Upper limit	Multiplicative model		Additive model	
					Lower limit	Upper limit	Lower limit	Upper limit
China	522	1630	1.67 (1.33, 2.09)	4.94 (2.45, 9.96)	0.00 (0.00, 0.00)	0.00 (0.00, 0.03)	0.00 (0.00, 0.00)	0.00 (0.00, 0.04)
Czechoslovakia	67	151	1.07 (1.04, 1.10)	1.16 (1.09, 1.24)	0.22 (0.18, 0.27)	0.29 (0.26, 0.31)	0.26 (0.22, 0.30)	0.31 (0.29, 0.32)
Colorado	35	104	1.03 (1.02, 1.05)	1.11 (1.06, 1.16)	0.36 (0.34, 0.39)	0.40 (0.39, 0.41)	0.40 (0.39, 0.41)	0.41 (0.41, 0.42)
Ontario	190	598	1.20 (1.11, 1.31)	1.80 (1.39, 2.32)	0.00 (0.00, 0.00)	0.19 (0.00, 0.48)	0.00 (0.00, 0.00)	0.23 (0.00, 0.54)
Newfoundland	58	173	1.06 (1.03, 1.08)	1.18 (1.10, 1.28)	0.60 (0.54, 0.67)	0.70 (0.68, 0.73)	0.71 (0.69, 0.73)	0.75 (0.74, 0.75)
Sweden	0	0	1.00 (1.00, 1.00)	1.00 (1.00, 1.00)	0.95 (0.95, 0.95)	0.95 (0.95, 0.95)	0.95 (0.95, 0.95)	0.95 (0.95, 0.95)
New Mexico	510	1618	1.65 (1.32, 2.05)	4.88 (2.43, 9.79)	0.00 (0.00, 0.18)	0.69 (0.38, 1.08)	0.00 (0.00, 0.43)	1.14 (0.77, 1.43)
Beaverlodge	0	0	1.00 (1.00, 1.00)	1.00 (1.00, 1.00)	2.21 (2.21, 2.21)	2.21 (2.21, 2.21)	2.21 (2.21, 2.21)	2.21 (2.21, 2.21)
Port Radium	6	17	1.01 (1.00, 1.01)	1.02 (1.01, 1.02)	0.18 (0.18, 0.18)	0.19 (0.19, 0.19)	0.18 (0.18, 0.19)	0.19 (0.19, 0.19)
Radium Hill	13	40	1.01 (1.01, 1.02)	1.04 (1.02, 1.06)	4.36 (4.06, 4.66)	4.82 (4.72, 4.93)	4.53 (4.29, 4.77)	4.89 (4.81, 4.96)
France	240	741	1.27 (1.14, 1.40)	2.07 (1.50, 2.84)	0.00 (0.00, 0.00)	0.00 (0.00, 0.11)	0.00 (0.00, 0.00)	0.00 (0.00, 0.12)

^a^All the negative values of the modified *β*_*radon*, *i*_ are substituted by zero.

^b^The modification is based on the values of *β*_*radon*, *i*_ in Table 1.

### Modification of the lung cancer risk from radon exposure in the 11 miner cohorts

We used two models to address the joint effects of multiple causes of disease: a multiplicative model and an additive model. In our study, the multiplicative model (Eq (4)) implies that the effect of radon exposure on lung cancer also depends on the effect of DEE exposure. In contrast, under the additive model (Eq (5)), the two effects are considered to be independent.

$$RR_{radon,modified}=RR_{radon}÷RR_{DEE}$$ (4)

$$RR_{radon,modified}=RR_{radon}-RR_{DEE}+1$$ (5)

To our knowledge, there is no epidemiologic or statistical evidence to adress the combined effects of the exposures to both radon and DEE directly. It is only possible to speculate on the potential combined effects of radon and DEE. Therefore, we applied analytic approaches to compare the combined effects based on either additivity or multiplicativity of the individual effects. If there are sub-multiplicative interactions between the two effects, such as smoking and radon [5], the modification effect will fall into the interval between additive scale and multiplicative scale.

The original ERR_radon_ in each cohort can be calculated by the simple linear model (Eq (1)), which rely on the *β*_*radon*, *i*_ and the mean radon exposure (Table 1). Then the multiplicative/additive models were used to derive the cohort-specific radon risks that modified by DEE exposures. The overall variations in the *β*_*radon*_ and ERR_radon_ were obtained through the linear regressions over the 11 miner cohorts. The corrected ERR_radon_ can then be used to modify the BEIR VI models (Eq (2)). All risk factors in the BEIR VI models are only related to the characteristics of the cohorts. Thus we assumed these risk factors would not be affected by the confounding with DEE exposures. Under this assumption, only the *β*_*radon*, *EAD(C)*_ were adjusted to modify the risks estimated by the BEIR VI models.

### EPA’s estimates

EPA used a single model to yield the geometrical mean of the results from the two BEIR VI models to arrive at a plausible estimate of indoor radon risk. They chose a scaled version of the EAC model as their risk model, see Eq (6).

$$ERR_{radon}=\beta_{EPA}\left(w_{5-14}+\theta_{15-24}w_{15-24}+\theta_{25+}w_{25+}\right)\emptyset_{age}\gamma_{z}$$ (6)

Where the *β*_*EPA*_ is adjusted to be 0.0634, as a scaled-down *β*_*radon*, *EAC*_ yielding the average risk prediction of the EAD/EAC models, by the geometrical mean of their coefficients. Therefore, the *β*_*EPA*_ can be re-calculated using the modified *β*_*radon*, *EAD*_ and *β*_*radon*, *EAC*_; the *γ*_*z*_ is always equal to 1 for the low residential concentration (< 0.5 WL; 50 pCi/L); the step function of the *φ*_*age*_ in the EAC model is discontinuous at ages 55, 65 and 75 y. Thus, EPA used splines to smooth the *φ*_*age*_ function to avoid such implausible discontinuities.

Based on this model, EPA used life-table methods to calculate the lifetime risks of lung cancer in the U.S. The age-specific baseline overall and lung cancer death rates for the general population are derived from the 1989–91 vital statistics and mortality data from the National Center for Health Statistics (NCHS). The effects of radon and cigarette smoking are strongly synergistic in causing lung cancer, since radon progeny can be attached to the particles in tobacco smoke and inhaled deeply into the lung. EPA assumed that the lung cancer death rates are 14 (males) or 12 (female) times greater for ever-smokers (ES) than never-smokers (NS). Then they proposed age-specific smoking prevalence data to obtain the lung cancer death rates for both ES and NS. When it comes to the risk modification by smoking, EPA applied a sub-multiplicative interaction between tobacco-smoking and exposure to radon progeny: the risk coefficients are 0.9*β*_*EPA*_ for ES and 2*β*_*EPA*_ for NS.

The EPA’s estimates mainly include lifetime risk per WLM (probability of radon-induced lung cancer death) and etiologic fraction (EF) (fraction of lung cancer deaths attributable to radon). The EF represents the fraction of lung cancer deaths in the exposed population in which radon played some causative role. The estimation is based on a lifetime exposure at an average U.S. residential radon level of 1.25 pCi/L, derived from the EPA’s National Residential Radon Survey [26]. An indoor concentration of 1 pCi/L would result in a radon progeny exposure rate of 0.144 WLM/y, assuming an equilibrium of 40% between radon and its progeny, and a home occupancy of 70% on average. Thus, the average indoor radon exposure for the U.S. population is 0.181 WLM/y. The gender- and smoking-specific estimations of the risk per WLM and the EF are calculated assuming stationary populations in which 53% of males and 41% of females are ES. A combined estimation for the entire population is calculated as a weighted average of the four stationary populations. The risk per WLM and the EF are weighted by the survival probabilities and the baseline lung cancer death rates, respectively. Moreover, the lifetime risk of lung cancer deaths by radon exposure can be calculated by the product of risk per WLM, average annual exposure rate and life expectancy (sum of survival probability per age). More detailed formulations can be find in EPA’s documentation [2].

## Results

### Modified lung cancer risk from radon in the 11 miner cohorts

We estimate that the overall radon exposure-response function (*β*_*radon*_) may be overestimated by 9% to 26% after accounting for exposure to diesel exhaust. The overall decline in the *β*_*radon*_ is assessed by the linear regression of the modified *β*_*radon*, *i*_ in each cohort. Table 2 lists the modified *β*_*radon*, *i*_ in the 11 miner cohorts by the *β*_*DEE*_ given by Vermeulen et al. [16] and estimated DEE dose range. The derivate lung cancer risk coefficients by radon decrease after considering the possible exposure to DEE, except the Sweden mine which we assumed did not use diesel equipment due to the years it was in operation. The *β*_*radon*, *i*_ decreases more with the modification by the multiplicative model (up to 26%) than by the additive model (up to 16%).

**Fig 1:**
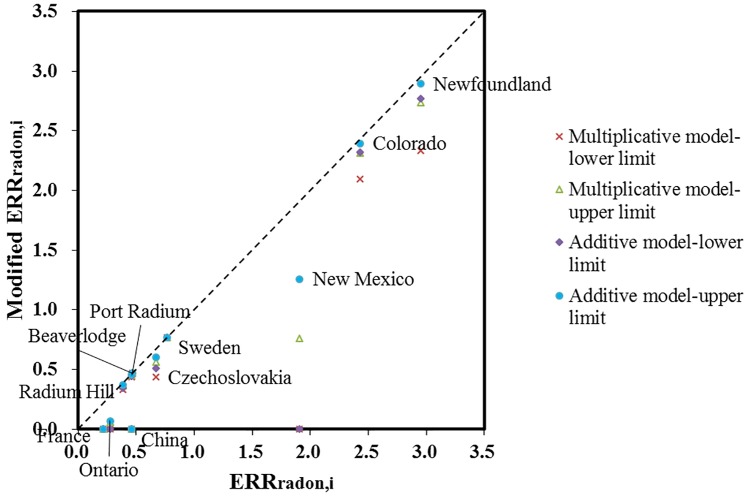
Scatter plot of the modified ERR_*radon*, *i*_ versus the original ERR_*radon*, *i*_ in each cohort.

The decline of the *β*_*radon*, *i*_ varies greatly across each cohort. Fig 1 shows the modifying effect on the ERR in each cohort, based on the results in Table 2. Generally, the excess risks of radon exposure drop more in the mines that had longer exposure duration and later first year exposed. This is attributable to the higher DEE exposure in those mines estimated from historic data. The estimated RR_DEE_ even exceeded the original RR_radon_ in China, Ontario and France miner cohorts. These three cohorts showed less potent exposure-response relationship with small magnitude of *β*_*radon*, *i*_ or mean radon exposure.

### Modified EPA’s estimates

Table 3 presents the modified EPA’s estimates of the risk per WLM and the EF. The calculated lifetime risks and EF are changed approximately proportional to the change of *β*_*EPA*_. The original risk estimate is 5.38 × 10^−4^ fatal lung cancer risks per WLM for the entire population. Our estimates of the modified risk per WLM (10^−4^) in the entire population ranged from 3.97 (95% CI: 3.65, 4.35) to 4.92 (95% CI: 4.79, 5.09). The estimated risk for ever smokers (ES) is approximately six times higher than for never smokers (NS).

**Table 3:** Modified EPA’s estimates of the risk per WLM and the EF.

Gender	Smoking Category	Risk per WLM (10^−4^) (95% CI)					Etiologic Fraction (95% CI)				
		EPA’s estimate	Multiplicative model		Additive model		EPA’s estimate	Multiplicative model		Additive model	
			Lower limit	Upper limit	Lower limit	Upper limit		Lower limit	Upper limit	Lower limit	Upper limit
Male	ES	10.60	7.82 (7.19, 8.56)	9.12 (8.75, 9.64)	8.85 (8.58, 9.28)	9.69 (9.45, 10.03)	0.129	0.095 (0.088, 0.104)	0.111 (0.106, 0.117)	0.108 (0.104, 0.113)	0.118 (0.115, 0.122)
	NS	1.74	1.28 (1.18, 1.41)	1.50 (1.44, 1.58)	1.45 (1.41, 1.52)	1.59 (1.55, 1.65)	0.279	0.206 (0.189, 0.225)	0.240 (0.230, 0.254)	0.233 (0.226, 0.244)	0.255 (0.249, 0.264)
	ES & NS	6.40	4.72 (4.34, 5.17)	5.51 (5.28, 5.82)	5.34 (5.18, 5.6)	5.85 (5.70, 6.05)	0.136	0.100 (0.092, 0.110)	0.117 (0.112, 0.124)	0.114 (0.110, 0.119)	0.124 (0.121, 0.129)
Female	ES	8.51	6.28 (5.78, 6.87)	7.32 (7.02, 7.74)	7.11 (6.89, 7.45)	7.78 (7.58, 8.05)	0.116	0.086 (0.079, 0.094)	0.100 (0.096, 0.105)	0.097 (0.094, 0.102)	0.106 (0.103, 0.110)
	NS	1.61	1.19 (1.09, 1.3)	1.39 (1.33, 1.46)	1.34 (1.3, 1.41)	1.47 (1.43, 1.52)	0.252	0.186 (0.171, 0.204)	0.217 (0.208, 0.229)	0.210 (0.204, 0.221)	0.230 (0.225, 0.238)
	ES & NS	4.39	3.24 (2.98, 3.55)	3.78 (3.62, 3.99)	3.67 (3.55, 3.84)	4.01 (3.91, 4.15)	0.131	0.097 (0.089, 0.106)	0.113 (0.108, 0.119)	0.109 (0.106, 0.115)	0.120 (0.117, 0.124)
Population	ES	9.68	7.14 (6.57, 7.82)	8.33 (7.99, 8.80)	8.08 (7.84, 8.47)	8.84 (8.63, 9.16)	0.124	0.092 (0.084, 0.100)	0.107 (0.102, 0.113)	0.104 (0.100, 0.109)	0.113 (0.110, 0.117)
	NS	1.67	1.23 (1.13, 1.35)	1.44 (1.38, 1.52)	1.39 (1.35, 1.46)	1.53 (1.49, 1.58)	0.263	0.194 (0.179, 0.212)	0.226 (0.217, 0.239)	0.220 (0.213, 0.230)	0.240 (0.234, 0.249)
	ES & NS	5.38	3.97 (3.65, 4.35)	4.63 (4.44, 4.89)	4.49 (4.36, 4.71)	4.92 (4.79, 5.09)	0.134	0.099 (0.091, 0.108)	0.115 (0.111, 0.122)	0.112 (0.108, 0.117)	0.122 (0.119, 0.127)

The EPA’s estimated etiologic fraction is 0.134 for the entire population, at a constant exposure rate of radon exposure of 0.181 WLM/y. The radon-induced lung cancer death fraction is approximately 1/8 for ES and 1/4 for NS. After the modification, we estimated that the EF of the entire population is reduced to up to 0.099 (95% CI: 0.091, 0.108) by the *β*_*DEE*_ of Vermeulen et al. [16]. In this situation, the radon exposure may only account for about 1/11 for ES lung cancer deaths and 1/5 for NS deaths.

The EF can also be multiplied by the lung cancer deaths in the entire population to obtain the total number of radon-induced lung cancer death. Here EPA used the lung cancer deaths data in 1995: 157,400 deaths from lung cancer (95,400 males and 62,000 females; 146,400 ES and 11,000 NS) [5]. EPA estimated that the number of radon-induced lung cancer deaths in 1995 was 21,100. With the modified EF from our analysis, the total number of the lung cancer deaths attributable to radon could be reduced by 1,800 (95% CI: 1,100, 2,300) to 5,500 (95% CI: 4,100, 6,800).

**Fig 2:**
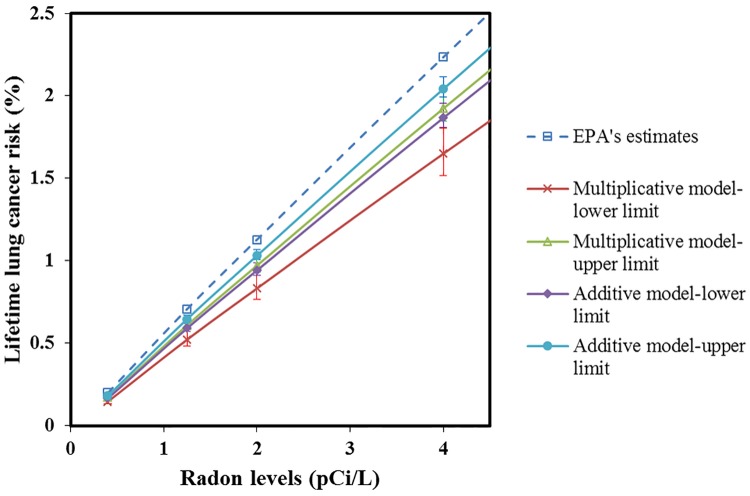
Modified EPA’s estimates of the lifetime lung cancer risks at various indoor radon exposure levels compared to EPA’s original estimate.

Fig 2 further shows the modified estimates of the lifetime lung cancer risk at various indoor radon exposure levels. The national average outdoor radon levels is 0.4 pCi/L, which is considered as the target indoor radon levels [27]. The estimated risk is relatively low at this level, but 2/3 of the U.S. homes exceed it. The 2 pCi/L is now the EPA’s “consider action” limit and is associated with an estimated lifetime risk of 1.20%. This could be reduced to up to 0.83% (95% CI: 0.77%, 0.91%) with the modification. At EPA’s required action level (4 pCi/L), EPA estimates that the lifetime lung cancer risk is 2.30% for the entire population. Our estimates indicate that the risk could be lowered to 1.65% (95% CI: 1.52%, 1.81%) at this level. That is roughly reduced by a concentration level of 1 pCi/L. The modified lifetime risks decrease more significantly with increasing radon levels.

## Discussion

The U.S. EPA’s estimate of 21,100 lung cancer deaths per year attributable to radon exposure in homes might be overestimated due to neglect the concurrent diesel exposure in miner studies upon which their risk estimates rely. We adjusted EPA’s risk models accounting for diesel exposure and estimate that radon accounts for 15,600 (95% CI: 14,300, 17,000)– 19,300 (95% CI: 18,800, 20,000) lung cancer deaths per year, a reduction of 9%–26% from EPA’s estimates. Further, EPA’s estimate of 21,100 deaths per year was based on the 1989–1991 vital statistics, which has not been updated since 2003. In 2014, there were a total of 155,528 deaths from lung cancer, including 84,861 males and 70,667 females [28]. Applying the age-specific mortality data in 2014 [28], EPA’s original models estimate that there are 17,400 deaths per year with an EF of 0.112, while our updated models estimate that the total number of deaths per year attributable to radon is between 12,900 (95% CI: 11,800, 14,100)%—15,900 (95% CI: 15,500, 16,500). The reduction may be as high as 40% when considering the updated mortality data. This is significant because although this maintains radon’s high public health importance as the second leading cause of lung cancer deaths in the U.S., it is becoming closer to secondhand smoking, which was ranked third (approximately 7,330 lung cancer deaths per year) [29].

Along with our findings that EPA’s estimates of radon risk are inflated after accounting for diesel, there is also other supporting evidence. Epidemiological studies in the general population were not included in the BEIR VI and EPA models. However, there is a growing body of data from case-control studies performed in homes directly. A recent review paper [30] reported that the odds ratios for lung cancer death varied from 0.7 to 4.2 at various indoor radon levels among 24 case-control studies. Sixteen studies showed an odds ratio greater than 1, yet only 7 out of 24 studies showed statistically significant associations, despite radon having one of the highest exposure-response functions (10^−3^ to 10^−4^ at EPA’s action level) for an environmental pollutant. The largest scale study they reviewed was a German research comprising 2,963 cases and 4,232 controls [9]. This study showed a solid linear dose-response relation. The overall EOR of lung cancer per 1 pCi/L was 0.037 (95% CI: -0.007, 0.111). A total of nine studies finally reported positive dose-response effects: ERR/EORs of lung cancer increase from 0.015 [31] to 0.104 [32] per a radon level of 1 pCi/L, compared to the EPA’s current age-specific estimates ranged from 0.009 (6 yrs.) to 0.282 (51 yrs.) per 1 pCi/L based on Eq (6). In addition to these studies, strong associations between residential radon exposure and lung cancer risk has also been observed by the combined analyses of seven primary North American case-control studies by Krewski et al. [10, 11]. But some other studies could not show persuasive evidences of the association between radon exposure and lung cancer [12–14, 33]. In general, the studies conducted at high radon levels tended to show strong associations [34, 35]. Those studies that failed to show a significant relation were usually performed in areas with relatively low radon concentration (< 100 Bq/m^3^) [13, 14, 33]. The inconsistencies across these population-based epidemiological studies may be due to radon being a less potent carcinogen than expected at low exposure level, or else we would expect to be able to detect persuasive and consistent associations in the general population. Additionally, the uncertainties regarding radon measurement [14] and selection bias among heavy smokers [12] may also lead to wrong estimation of exposure-response relationship. Further residential studies are suggested to include large samples of both smokers and non-smokers, precise and long-term radon concentration measurement, and wide range of indoor radon exposure.

Our study on indoor radon risks was designed to produce more robust estimates of lung cancer deaths attributable to indoor radon exposure on a population scale, thus informing radon mitigation policies. The attributable risk (AR) is a common-used concept to evaluate the potential reduction of the incidence if the exposure is eliminated [5]. Kim et al. [36] summarized the ARs for radon ranged from 0.033 (UK) to 0.200 (Sweden) worldwide with mean indoor radon concentrations from 0.6 to 3 pCi/L. The estimates of AR vary considerably based on the risk models used [37] and mean indoor radon concentrations. In the U.S., the overall AR was estimated to be 0.139 with the EAC model and 0.098 with the EAD model [5] at a mean radon level of 1.25 pCi/L. Alternatively, EPA reported EF instead of AR with a scaled EAC model. Their original estimate (0.134) is slightly lower than that given by the EAC model. Otherwise, the EF would be in the range from 0.017 to 0.117, estimated by the constant risk models (risk increases from 0.015 to 0.104 per 1 pCi/L) from nine positive case-control studies [30]. Specifically, the EF would be 0.067 estimated by the constant risk model (ERR increases 0.16 per 100 Bq/m^3^, corresponding to 0.060 per 1 pCi/L) from 13 European case-control studies [8]. All the EFs estimated by the risk models from these residential studies are lower than current EPA’s estimate. This may partly imply the radon risks are overestimated using the EPA’s risk model. In our study, the modified EF ranged from 0.099 to 0.122 (causing 15,600 to 19,300 deaths) as shown in Table 3, an estimate that is between the U.S. and European estimates, which is more comparable to the estimates based on residential studies.

Uncertainties associated with miner studies from using miner data and extrapolating to the general population is likely to further contribute to inaccurate estimations of risks attributable to radon beyond our accounting for diesel exposure in this study [5]. The miner data is subject to errors in exposure ascertainment and information on miners’ vital status. There is also limited data on other confounding agents, such as smoking, diesel, arsenic and silica. Another critical issue is that the direct extrapolation of the miners to the general population fails to account for several key factors. First, the two populations have significant differences in sex & age distributions and smoking prevalence; the miners were all adult men with a high prevalence of smokers and therefore the radon dose may be affected by the differences in physical and physiological factors, such as breathing rates and bronchial configurations. Second, the cumulative radon exposures in the mine were often 10 times higher than the lifetime exposures that are encountered in homes. In the BEIR VI and EPA models, the indoor radon risks are linearly extrapolated from the miner risks without a threshold. The BEIR VI committee recognized that their understanding of this assumption was incomplete. The evidence for the linear-non-threshold hypothesis is not determinative. Duan et al. [38] found evidence of a non-linear dose-response relationship through a meta-analysis of published cohort and case-control studies. They reported that the non-linearity was more obvious when the radon level was higher than 200 Bq/m^3^. The lung cancer risk was approximately linearly related to residential radon exposure below this level. Therefore, EPA could also consider incorporating recent epidemiological analyses of linear dose-response relation at residential exposures into their risk assessment, instead of fully dependent on downward extrapolation from the high-dose miner data.

Our study has limitations on DEE risk estimations. Diesel exposure measurements were not made for the 11 miner studies, requiring us to estimate exposures in these mines. We examined the DEE risks with a plausible diesel exposure range based on the historical data [17, 23, 39] in other mines that were operating at the same time as the 11 miner studies. In these studies, carbon monoxide (CO) was used as a surrogate for REC. In addition, because CO samples were limited in earlier years, they used the ratio of diesel engine horsepower to the mine air exhaust rate to estimate CO level. Although the surrogate approach was largely limited by the data available, the significant uncertainties in REC estimates need to be further investigated. Recently, an industry trade group published another study focusing on the DEE risk estimation [40]. In their evaluation, the meta-analysis could decrease to up to 10%-20% of the primary value published by Vermeulen et al. [16]. Their optimal fitting of *β*_*DEE*_ is 0.00032 (95% CI: 0.00002, 0.00062) with a p-value of 0.035. Applying this value of *β*_*DEE*_, the overall *β*_*radon*_ would be decreased by 3%%–14%, as opposed to 9%%–26% using the results from Vermeulen et al. [16] (Fig A in S1 File). The corresponding modifications are presented in Figs B and C, and Tables A and B in S1 File. As a result, the number of radon-induced lung cancer deaths could be reduced by 600 (95% CI: 0, 1,300) to 2,900 (95% CI: 200, 4,400) in the general population. The EPA’s death estimates still decreased a lot with this alternative modifying approach.

## Conclusions

EPA’s original estimates of fatal risks attributable to radon may be overestimated by 9%– 26%, after accounting for exposure to diesel in the miner studies originally used to estimate radon risk. Our best estimates, using updated models and mortality data from 2014, indicate that there could possibly be 12,900–15,900 deaths attributable to indoor radon exposure each year. Even with these modified risks attributable to radon, overall radon risk is still elevated above ‘acceptable’ levels relative to other environmental hazards. Our results do not argue for modifying the current action levels. We recommend EPA further re-evaluate the health effects of exposure to radon that account for new information on diesel exhaust carcinogenicity in BEIR VI models, up-to-date vital statistics and new epidemiological evidences from residential studies.

## Supporting information

S1 File Radon diesel and lung cancer. (DOC)
