# Supplementary material for: Radon-induced lung cancer deaths may be overestimated due to failure to account for confounding by exposure to diesel engine exhaust in BEIR VI miner studies
Source: PLoS One. 2017 Sep 8;12(9):e0184298. doi: 10.1371/journal.pone.0184298 (PMC5590909; doi:10.1371/journal.pone.0184298)
Supplement: S1 File — (DOC) [file pone.0184298.s001.doc]

**Supporting information**

**Radon-induced lung cancer deaths may be overestimated due to failure to account for** **confounding by exposure to diesel engine exhaust** **in BEIR VI miner studies**

*Xiaodong Cao, Piers MacNaughton, Jose Cedeno Laurent, Joseph G. Allen*

Department of Environmental Health, Harvard T.H. Chan School of Public Health, Boston, MA 02215, USA

**Table of contents**

Supporting information, **Table A**, page 2

Supporting information, **Table B**, page 3

Supporting information, **Fig A**, page 4

Supporting information, **Fig B**, page 4

Supporting information, **Fig C**, page 5

**Table A. Modified mean *β_radon,i_* in the 11 miner cohorts by the *β_DEE_* of** [**Morfeld and Spallek (2015)**](#_ENREF_11)**.**

| Cohort | DEE dose (μg/m^3^-years) | | RR_DEE_ (95% CI) | | Modified mean *β_radon,i_* (%) (95% CI) ^a, b^ | | | |
| --- | --- | --- | --- | --- | --- | --- | --- | --- |
|  | Lower limit | Upper limit | Lower limit | Upper limit | Multiplicative model | | Additive model | |
|  |  |  |  |  | Lower limit | Upper limit | Lower limit | Upper limit |
| China | 522 | 1630 | 1.18 (1.01, 1.38) | 1.68 (1.03, 2.75) | 0.00 (0.00, 0.14) | 0.08 (0.02, 0.15) | 0.00 (0.00, 0.15) | 0.10 (0.03, 0.16) |
| Czechoslovakia | 67 | 151 | 1.02 (1.00, 1.04) | 1.05 (1.00, 1.10) | 0.30 (0.26, 0.34) | 0.32 (0.31, 0.34) | 0.31 (0.29, 0.34) | 0.33 (0.32, 0.34) |
| Colorado | 35 | 104 | 1.01 (1.00, 1.02) | 1.03 (1.00, 1.07) | 0.40 (0.38, 0.42) | 0.41 (0.41, 0.42) | 0.41 (0.41, 0.42) | 0.42 (0.42, 0.42) |
| Ontario | 190 | 598 | 1.06 (1.00, 1.12) | 1.21 (1.01, 1.45) | 0.17 (0.00, 0.84) | 0.65 (0.43, 0.87) | 0.21 (0.00, 0.85) | 0.69 (0.49, 0.88) |
| Newfoundland | 58 | 173 | 1.02 (1.00, 1.04) | 1.06 (1.00, 1.11) | 0.71 (0.66, 0.76) | 0.74 (0.72, 0.76) | 0.75 (0.73, 0.76) | 0.76 (0.75, 0.76) |
| Sweden | 0 | 0 | 1.00 (1.00, 1.00) | 1.00 (1.00, 1.00) | 0.95 (0.95, 0.95) | 0.95 (0.95, 0.95) | 0.95 (0.95, 0.95) | 0.95 (0.95, 0.95) |
| New Mexico | 510 | 1618 | 1.18 (1.01, 1.37) | 1.68 (1.03, 2.73) | 0.66 (0.06, 1.64) | 1.33 (1.01, 1.69) | 1.11 (0.16, 1.69) | 1.56 (1.38, 1.71) |
| Beaverlodge | 0 | 0 | 1.00 (1.00, 1.00) | 1.00 (1.00, 1.00) | 2.21 (2.21, 2.21) | 2.21 (2.21, 2.21) | 2.21 (2.21, 2.21) | 2.21 (2.21, 2.21) |
| Port Radium | 6 | 17 | 1.00 (1.00, 1.00) | 1.01 (1.00, 1.01) | 0.19 (0.18, 0.19) | 0.19 (0.19, 0.19) | 0.19 (0.19, 0.19) | 0.19 (0.19, 0.19) |
| Radium Hill | 13 | 40 | 1.00 (1.00, 1.01) | 1.01 (1.00, 1.03) | 4.83 (4.61, 5.05) | 4.98 (4.91, 5.06) | 4.89 (4.73, 5.05) | 5.00 (4.95, 5.06) |
| France | 240 | 741 | 1.08 (1.00, 1.16) | 1.27 (1.01, 1.58) | 0.00 (0.00, 0.33) | 0.21 (0.08, 0.35) | 0.00 (0.00, 0.33) | 0.23 (0.09, 0.35) |

^a^All the negative values of the modified mean *β_radon,i_* are substituted by zero.

^b^The modification is based on the mean values of *β_radon,i_* in Table 1.

**Table B. Modified EPA’s estimates of the risk per WLM and the EF by the *β_DEE_* of** [**Morfeld and Spallek (2015)**](#_ENREF_11).

| Gender | Smoking Category | Risk per WLM (10^-4^) (95% CI) | | | | | Etiologic Fraction (95% CI) | | | | |
| --- | --- | --- | --- | --- | --- | --- | --- | --- | --- | --- | --- |
|  |  | EPA’s estimate | Multiplicative model | | Additive model | | EPA’s estimate | Multiplicative model | | Additive model | |
|  |  |  | Lower limit | Upper limit | Lower limit | Upper limit |  | Lower limit | Upper limit | Lower limit | Upper limit |
| Male | ES | 10.6 | 9.14 (8.40, 10.48) | 10.02 (9.53, 10.56) | 9.69 (9.13, 10.54) | 10.28 (9.94, 10.58) | 0.129 | 0.111 (0.102, 0.128) | 0.122 (0.116, 0.129) | 0.118 (0.111, 0.128) | 0.125 (0.121, 0.129) |
|  | NS | 1.74 | 1.50 (1.38, 1.72) | 1.64 (1.56, 1.73) | 1.59 (1.50, 1.73) | 1.69 (1.63, 1.74) | 0.279 | 0.241 (0.221, 0.276) | 0.264 (0.251, 0.278) | 0.255 (0.240, 0.277) | 0.271 (0.262, 0.279) |
|  | ES & NS | 6.40 | 5.52 (5.07, 6.33) | 6.05 (5.75, 6.38) | 5.85 (5.51, 6.37) | 6.21 (6.00, 6.39) | 0.136 | 0.117 (0.108, 0.134) | 0.129 (0.122, 0.136) | 0.124 (0.117, 0.135) | 0.132 (0.128, 0.136) |
| Female | ES | 8.51 | 7.34 (6.75, 8.42) | 8.04 (7.65, 8.48) | 7.78 (7.33, 8.46) | 8.26 (7.98, 8.50) | 0.116 | 0.100 (0.092, 0.115) | 0.110 (0.104, 0.116) | 0.106 (0.100, 0.115) | 0.113 (0.109, 0.116) |
|  | NS | 1.61 | 1.39 (1.28, 1.59) | 1.52 (1.45, 1.60) | 1.47 (1.39, 1.60) | 1.56 (1.51, 1.61) | 0.252 | 0.217 (0.200, 0.249) | 0.238 (0.226, 0.251) | 0.230 (0.217, 0.251) | 0.244 (0.236, 0.252) |
|  | ES & NS | 4.39 | 3.78 (3.48, 4.34) | 4.15 (3.95, 4.37) | 4.01 (3.78, 4.37) | 4.26 (4.12, 4.38) | 0.131 | 0.113 (0.104, 0.13) | 0.124 (0.118, 0.131) | 0.120 (0.113, 0.13) | 0.127 (0.123, 0.131) |
| Population | ES | 9.68 | 8.35 (7.68, 9.57) | 9.15 (8.70, 9.64) | 8.85 (8.34, 9.63) | 9.39 (9.08, 9.66) | 0.124 | 0.107 (0.098, 0.123) | 0.117 (0.111, 0.124) | 0.113 (0.107, 0.123) | 0.120 (0.116, 0.124) |
|  | NS | 1.67 | 1.44 (1.32, 1.65) | 1.58 (1.50, 1.66) | 1.53 (1.44, 1.66) | 1.62 (1.57, 1.67) | 0.263 | 0.227 (0.209, 0.260) | 0.249 (0.236, 0.262) | 0.240 (0.227, 0.262) | 0.255 (0.247, 0.263) |
|  | ES & NS | 5.38 | 4.64 (4.27, 5.32) | 5.08 (4.83, 5.36) | 4.92 (4.63, 5.35) | 5.22 (5.05, 5.37) | 0.134 | 0.116 (0.106, 0.133) | 0.127 (0.120, 0.134) | 0.122 (0.115, 0.133) | 0.130 (0.126, 0.134) |


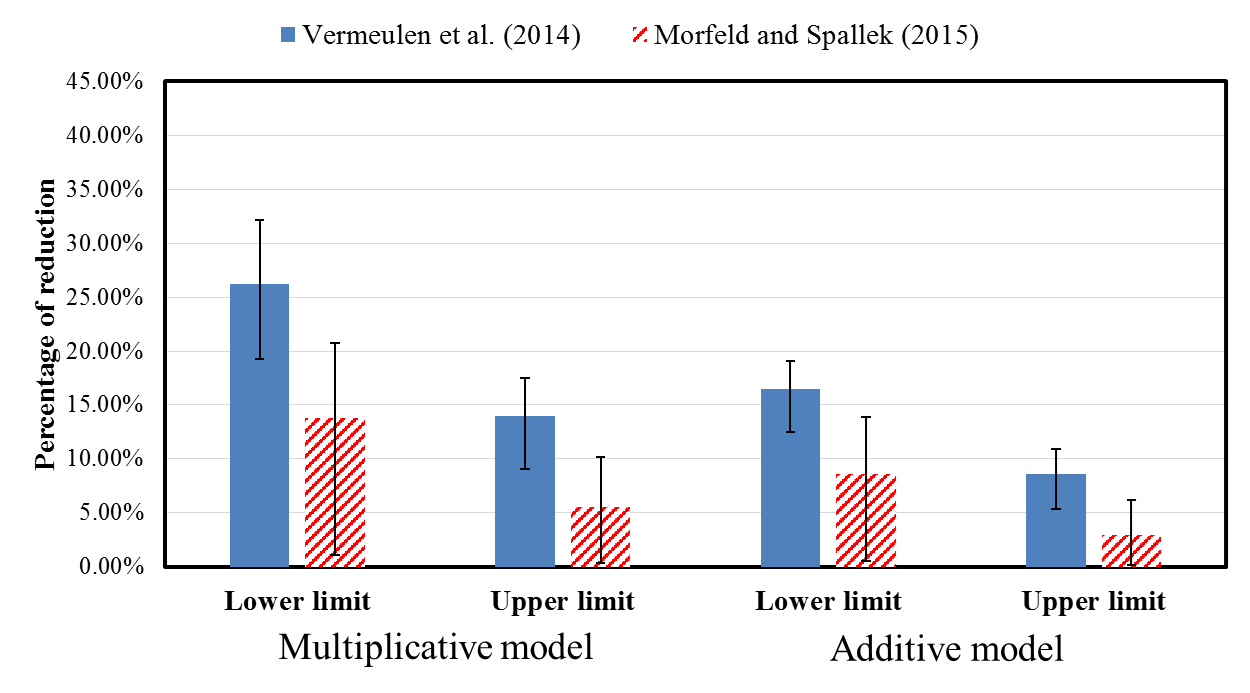


**Fig A. The reduction of the modified overall *β_radon_* using two different estimates of the exposure-response function for diesel exhaust.**

**Fig B. Scatter plot of the modified ERR*_radon,i_* versus the original ERR*_radon,i_* in each cohort with the *β_DEE_* of** [**Morfeld and Spallek (2015)**](#_ENREF_11)**.**

**Fig C.** **Modified EPA’s estimates of the lifetime lung cancer risk at various indoor radon exposure levels with the *β_DEE_* of** [**Morfeld and Spallek (2015)**](#_ENREF_11)**.**
